# Cloning and characterization of an ABA-independent DREB transcription factor gene, *HcDREB2*, in *Hemarthria compressa*

**DOI:** 10.1186/s41065-016-0008-y

**Published:** 2016-04-08

**Authors:** Yongxia Chen, Linkai Huang, Haidong Yan, Xinquan Zhang, Bin Xu, Xiao Ma

**Affiliations:** 1 Animal Science Department, Xichang college, Xichang, 615000 China; 2 Grassland Science Department, Sichuan Agricultural University, Ya’an, 625014 China; 3 College of Agro-grassland Science, Nanjing Agricultural University, Nanjing, 210095 China

**Keywords:** Cold stress, DREB transcription factor, Drought and salt stress, *Hemarthria compressa*, Yeast one-hybrid system

## Abstract

**Background:**

*Hemarthria compressa* is a stoloniferous perennial tropical forage grass with a wide geographic distribution; however, environmental stress has a great influence on its growth. The DREB transcription factor family genes contains candidate genes for improving plant stress tolerance.

**Results:**

From cold-treated *H. compressa* plants, a putative *DREB2* gene (*HcDREB2*) was cloned using the RACE-PCR method. *HcDREB2* was 1296 bp in length and encoded a putative protein 264 amino acid residues long. *HcDREB2* shared the highest sequence identity with *DREB2* in sorghum. The expression of HcDREB2 was independent of ABA treatment, but inducible by low temperatures as well as drought and high salinity treatments. Yeast one-hybrid assays showed that HcDREB2 directly bound the DRE cis-acting element to transactivate the expression of the downstream reporter genes.

**Conclusions:**

HcDREB2, a stress-inducible but ABA-independent transcription factor gene, can transactivate downstream genes by binding to the DRE cis-element. The current results are a foundation for making use of this stress tolerance gene in future *H. compressa* studies.

**Electronic supplementary material:**

The online version of this article (doi:10.1186/s41065-016-0008-y) contains supplementary material, which is available to authorized users.

## Background

*Hemarthria compressa*, commonly called whip grass, is a perennial grass in the family Gramineae. It is mainly distributed in tropical and subtropical zones, with a sparse distribution in the temperate-humid zones of the Northern Hemisphere. *H. compressa* is characterized by its long growing period, high growth rate, strong regenerative capacity, high yield, and strong stress tolerance. In southern China, *H. compressa* is an important forage grass, and is often used for soil and water conservation as well as ecological management [1, 2]. Most *H. compressa* studies have focused on agronomic characters, production performance, and genetic diversity. Currently, genetic information about *H. compressa* is very rare, and no sequence records of this important forage grass have been available in the NCBI GenBank until now. This limits research on functional genes, but transcription factors, as regulatory proteins that regulate signal transduction and gene expression, play an especially important role in exploring gene function. Therefore, identification and characterization of transcription factor will contribute to gene functional studies in *H. compressa* and other grasses.

Transcription factors are DNA-binding proteins that can specifically bind to one or more cis-acting elements in the promoters of eukaryotic genes, thereby activating or inhibiting transcription. Based on conserved DNA domains, transcription factors are classified into various families such as AP2/ERF, MYB, WRKY, and bZIP. [1, 2]. AP2/ERF is a unique transcription factor family found in plants; it participates in plant development [3–6], hormonal (ABA, ethylene, and others) responses, secondary metabolism, and responses to biotic or abiotic stresses [7, 8]. Based on similarities found in their AP2/ERF domains, Sakuma et al. [9] divided the transcription factors encoding the AP2/ERF domain in *Arabidopsis* into five groups: four sub-families (AP-2, RAV, DREB, and ERF) and the remaining transcription factors.

The DREB (dehydration responsive element binding protein) subfamily genes play key roles in plant stress responses to low temperatures, drought, and high salinity environments [7, 10, 11]. DREB has a conserved AP2/ERF domain that can specifically bind to the DRE/CRT cis-acting element to activate expression of several stress-tolerance genes, thereby enhancing plant stress tolerance [7, 11, 12]. In *Arabidopsis*, DREB1A, DREB1B, and DREB1C play significant roles in cold-responsive gene expression. With respect to its AP2/EREBP domain, CBF4/DREB1D displays high homology with DREB1A; however, the expression of CBF4/DREB1D was induced by osmotic stresses instead of cold stress [9, 13]. DREB2A and DREB2B were contained in plants exposed to salinity or drought stresses, while DREB2C, DREB2D, and DREB2F were slightly induced in leaves by salinity treatment while DREB2E was slightly induced in roots by exogenous ABA treatment [9, 10].

In recent years, DREB transcription factors have been identified in a large number of model plant species, including *Nicotiana tabacum* [14], *Arabidopsis thaliana* [9], and rice (*Oryza sativa*) [15],. DREB genes have been studied in forage crops as well. For example, Tang et al. [16] isolated a new DRE-binding protein gene, FaDREB1, from *Festuca arundinacea* using the rapid amplification of cDNA end-PCR (RACE-PCR) method, which discovered an AP2/ERFBP-type transcription factor and found that the FaDREB1 protein may be involved in the regulation of cold stress via the ABA-independent pathway. Xiong et al. [17] identified a DREB1A/CBF3-like gene *LpCBF3* from perennial ryegrass (*Lolium perenne* L.) and demonstrated that LpCBF3 is similar to DREB/CBF genes in *Arabidopsis* and is a functional transcription regulator in *Arabidopsis*. In alfalfa (*Medicago falcata* L.), two DRE-binding protein genes, *MfDREB1s* and *MfDREB1*, which encode AP2/EREBP-type transcription factors, were both found to be inducible by low-temperature stress [18]. Yet, the DREB family of genes in warm-season perennial grasses has been poorly examined so far.

In this study, we used RACE-PCR to clone DREB transcription factor genes from *H. compressa*. Using to the conserved sequence of the AP2/ERF domain, we successfully cloned a full-length HcDREB2 gene from *H. compressa*. The HcDREB2 protein bound DRE cis-elements to transactivate the reporter gene in the yeast one-hybrid assay. Moreover, the expression level of HcDREB2 was highly induced by various stress treatments but not by ABA. No DREB genes were identified in *H. compressa*, and these experimental results provide the first valuable genetic information about the DREB gene family in this species. These results provide a foundation for stress-tolerance breeding in *H. compressa* through genetic engineering.

## Results and discussion

### Isolation of HcDREB

**Fig. 1:**
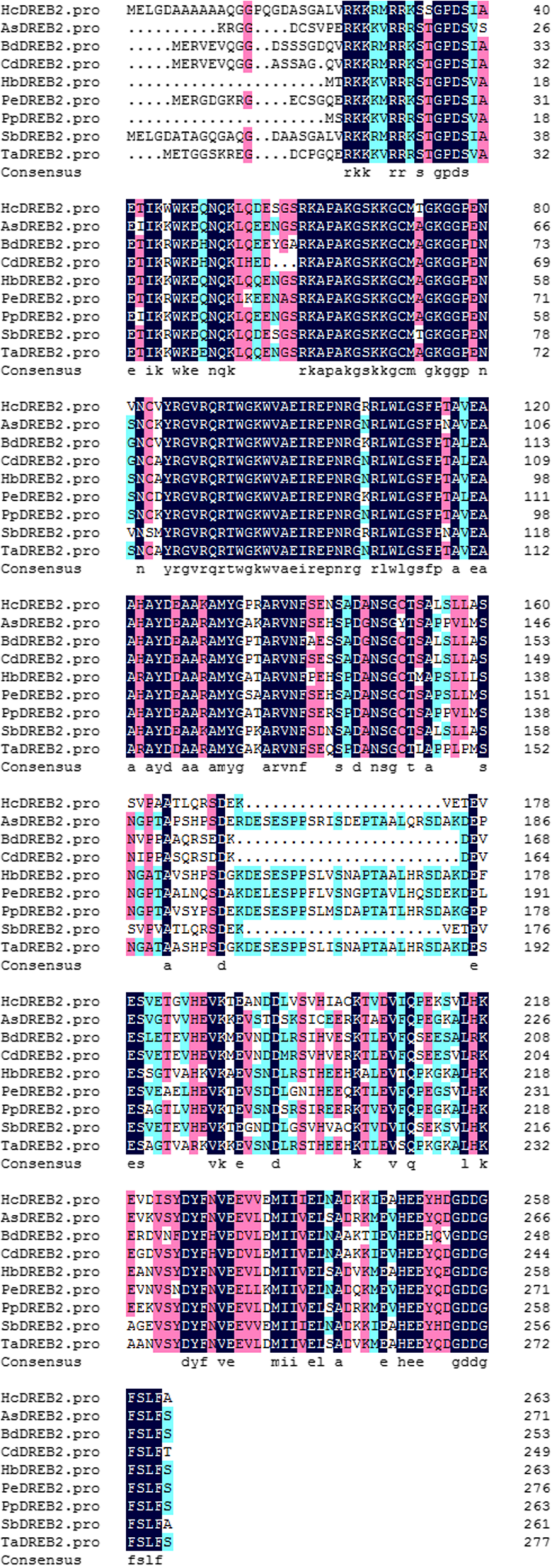
Multiple sequence alignment of AP2/EREBP domains. AsDREB2 (GenBank ID: EF672101), *Avena sativa*; BdDREB2 (EF512460), *Buchloe dactyloides*; CdDREB2 (AY462118), *Cynodon dactylon*; HbDREB2 (AY728807), *Hordeum brevisubulatum*; PeDREB2 (EU295482), *Phyllostachys edulis*; PpDREB2 (AY553331), *Poa pratensis* putative; SbDREB2 (DQ403725), *Sorghum bicolor*; TaDREB2 (AF303376.1), and *Triticum aestivum*. Identical amino acid residues are shaded in color (dark blue indicate identical residues while light blue and red similar residues)

A full-length HcDREB gene, HcDREB2 (GenBank ID: KC203598.1), was isolated from cold-treated H. compressa samples. The gene is 1254 bp in length and encodes a putative polypeptide of 264 amino acids (Additional file 1: Table S1). Although the full sequence of HcDREB shared only moderate identity with known DREB proteins in other plant species, it contained a conserved AP2/EREBP domain, including two highly conserved residues (V14 and E19) that are critical to its interaction with the DRE cis-element. This region (between V14 and E19) is unique to DREB2 transcription factors. Besides these conserved residues and domain, we also observed two additional features that are common to DREB proteins: (1) the nucleic localization signal region on the N terminus, which is rich in alkaline amino acids and (2) a transcriptional activation region that is rich in acidic amino acids (Fig. 1). These observations provide additional evidence that the sequence we obtained encoded a DREB family gene.

### Expression analysis of HcDREB2

**Fig. 2:**
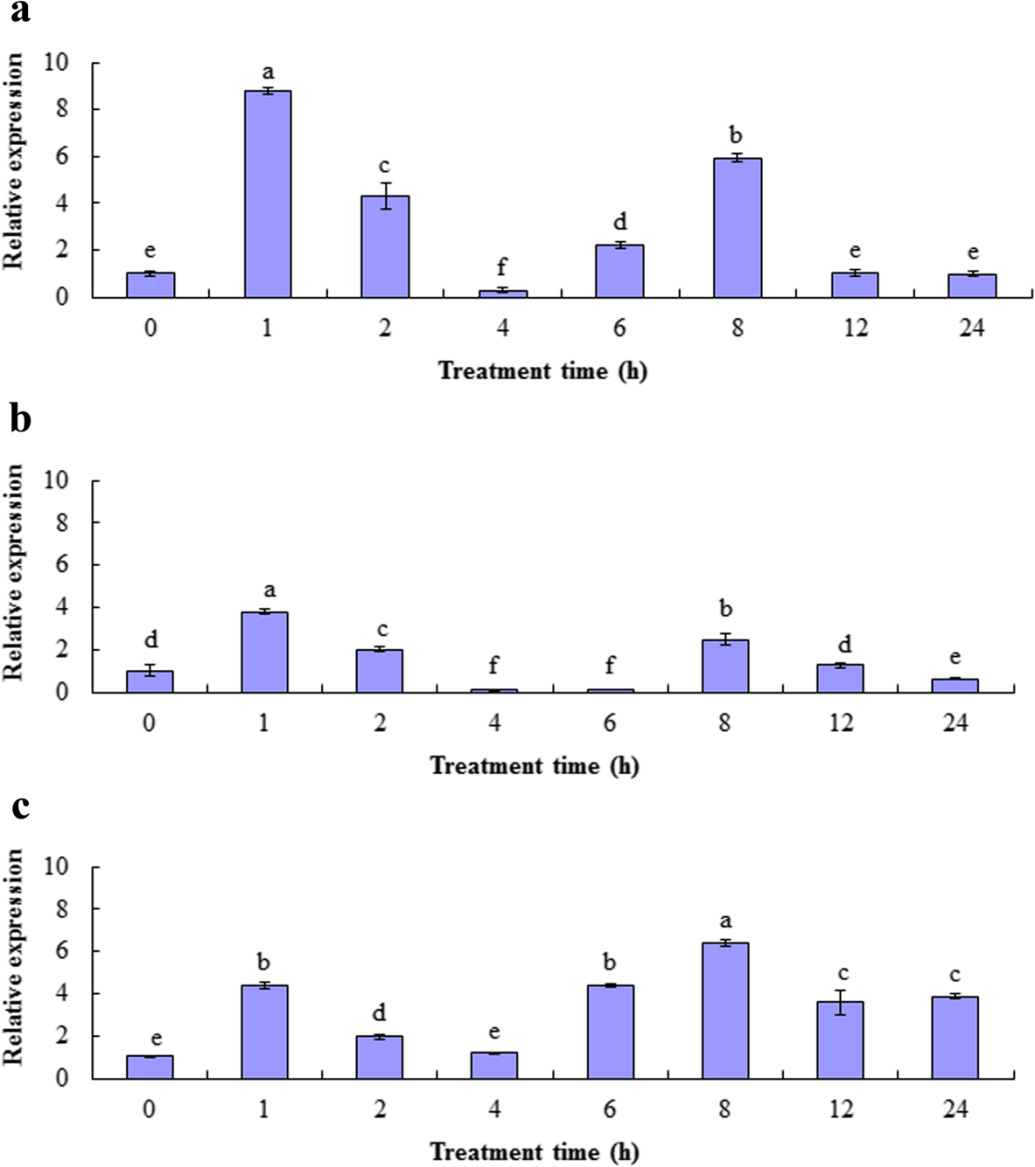
The relative quantification of HcDREB2 under low-temperature, drought, and salinity treatments was determined by qRT-PCR using one reference gene (β-actin). Expression pattern of HcDREB2 are shown under **a** low-temperature treatments, **b** drought stress, and **c** high salinity stress. The error bars indicate the standard error (SE) of three biological replicates. Different letters on the adjacent columns indicate significant differences (*P* < 0.05)

The relative transcription levels of HcDREB2 under low temperature, drought, high salinity, and ABA treatments were determined by qRT-PCR (Fig. 2). HcDREB2 expression was not induced by ABA treatment and remained at a level that was barely detectable by qRT-PCR (Ct value >32). On the contrary, the expression level of HcDREB2 was quickly induced by the other stress treatments. Two peak values were observed during the time course of the treatments; the first and the second peaks were observed after 1 and 8 h, respectively, of cold, drought, and salinity treatments (Fig. 2). After 24 h of treatment, the HcDREB2-expression level declined under cold and drought treatments, but remained at a relatively high level under the salinity treatment (Fig. 2). In brief, the expression of HcDREB2 was independent of ABA but inducible by the three tested abiotic stresses.

It has been suggested that the DREB2 transcription factor participates in stress responses to drought and high NaCl concentration. The expression of DREB2 genes in *Arabidopsis* and rice were induced by drought and high NaCl concentrations but not low temperatures [10, 19]. Our studies indicate that HcDREB2 gene expression is induced by exposing plants to low temperatures. It is known that the DREB2 gene in some plants can regulate heat-shock response and activate the expression of many abiotic stress genes. The expression of DvDREB2A in chrysanthemum was induced by high temperatures [20]. Over-expression of the ZmDREB2A gene strengthens the tolerance of Arabidopsis for exposure to high temperatures [21]. It has been shown that AtDREB2A, the close homolog of HcDREB2 in *Arabidopsis*, was induced by drought and high NaCl concentration exposure and was also involved in the penetration stress signal transduction pathway. AtDREB2A went through post-translational modification, and the activated form regulated the expression of a series of down-stream functional genes with DRE elements in their promoters [22, 23]. Despite the similarities, the expression modes of DREB2 genes differed across gene family members and varied across different plant species. The expression mode of DREB genes might also lead to their diversified gene functions by targeting and regulating the downstream functional genes thereby contributing to varied stress tolerances in plants. For examples, TaDREB1 was induced by low temperatures of 0 °C with a photoperiod of 12 h, 20 % PEG6000 for drought stress, 200 mM NaCl for salt stress, and 25 mM ABA for ABA stress in different wheat (*Triticum aestivum*) cultivars [24]; HvDRF1 was induced by drought achieved by water deprivation of 3-week-old plants grown in small pots containing sandy soil as well as salinity and ABA treatments that were performed by the hydroponic growth of barley seedlings (*Hordeum vulgare*; 9-day-old plants) in 100 μm ABA or 250 mm NaCl solution for 6 h [25]. The transcriptional expression of PgDREB2A in *Pennisetum laucum* is induced by low temperatures and salinity as shown by an experiment conducted with 12-day-old seedlings that were subjected to salt stress (250 mM NaCl) and cold stress (4 °C) for 6, 12, 24, and 48 h, and drought stress that was achieved by drying plants on tissue paper and keeping them wrapped in dry tissue paper for 1 and 2 h [26]. In this study, we showed that HcDREB2 in *H. compressa* was induced by low temperatures and drought, but not by ABA, which implies that HcDREB2 might play an important role in the stress response of *H. compressa* and that HcDREB2-mediated stress tolerance might be ABA-independent.

The time course of DREB2 expression in response to abiotic stresses also differed among species. For example, under drought and salinity conditions for merely 10 min, AtDREB2 expression was upregulated quickly in Arabidopsis and reached peak expression after 10 h of treatment [10]. The expression of BeDREB2 in Bermuda grass (*Cynodon dactylon*) reached its peak when the plant was exposed to salinity for 4 h [27]. In *Malus robusta*, DREB2 expression peaked when exposed to either low temperatures for 8 h or drought for 1 h [28]. In contrast, in *H. compressa*, the expression levels of HcDREB2 were lowest under low-temperature, drought, or salinity treatments for 4 h. Specifically, the expression levels of HcDREB2 under low temperatures and drought treatments for 4 h were even below those of the control. The expression of HcDREB2 was induced by low temperatures, drought, and high NaCl concentration and peaked twice (at 1 h and 8 h after treatment) over the 24-h period. This fluctuation of gene expression might reflect adaptation to photoperiod or its circadian rhythm. Fowler et al. [29] found that under low temperatures the expression of CBF3 was regulated by a photo signal, which participated in the related-cell signaling pathway by means of rhythm regulation and energy balance (through photosynthesis). Further studies examining whether the photoperiod regulated the induction of HcDREB2 expression are important for understanding this phenomenon in *H. compressa*.

### Yeast one-hybrid experiment

**Fig. 3:**
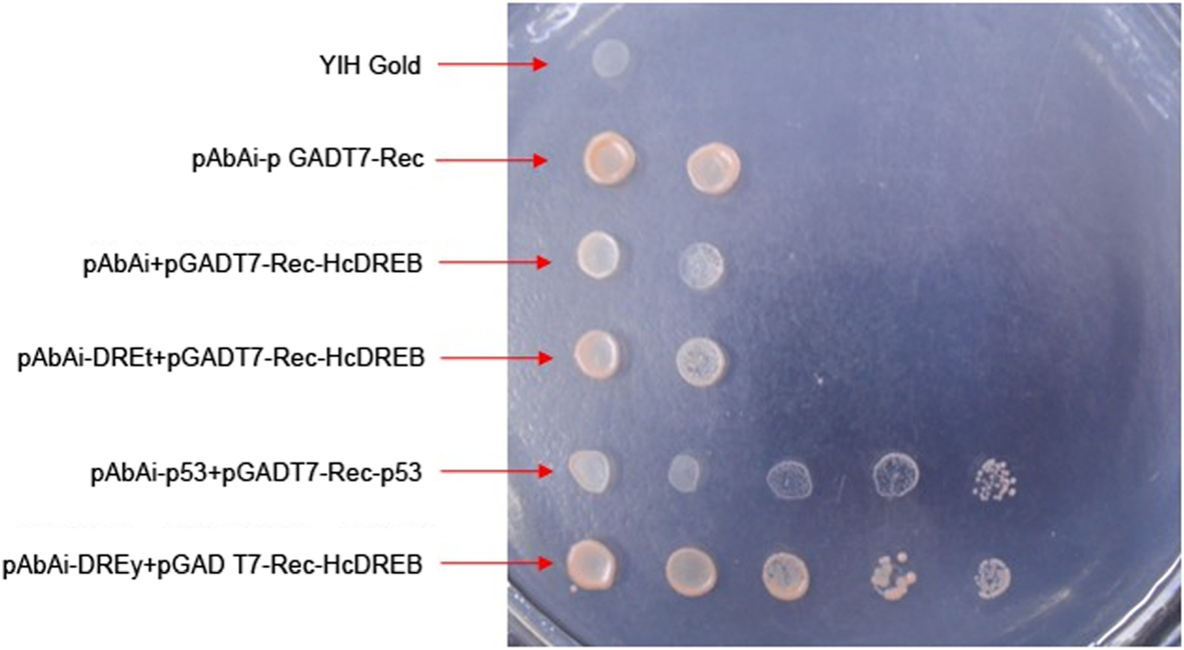
Trans-activation of dual reporter genes in yeast by the protein encoded by HcDREB2. The columns of colonies were transformed by the following constructs (from top to bottom): YIH Gold; pAbAi + pGADT7-Rec; pAbAi + pGADT7-Rec-HcDREB; pAbAi-DREt + pGADT7-Rec-HcDREB; pAbAi-p53 + pGADT7-Rec-p53; and pAbAi-DREy + pGADT7-Rec-HcDREB

A yeast one-hybrid assay was conducted to evaluate the transcriptional activation of HcDREB2. We observed that yeast cells transformed with pAbAi-p53 + pGADT7-Rec-p53 (positive control cells) and pGADT7-Rec-HcDREB + pAbAi-DREy were able to grow on SD-screening medium. In contrast, cells transformed with pAbAi + pGADT7-Rec, pGADT7-Rec-HcDREB + pAbAi-DREt, or pAbAi + pGADT7-Rec-HcDREB were unable to grow (Fig. 3). This result suggests that HcDREB2 directly binds DRE cis-elements and trans-activates the transcription of downstream genes.

Compared with DREB1 transcription factors, studies on DREB2 transcription factors are less common, probably because DREB2 transcription factors bind to DRE elements, but only induce low transcription activity. In a transcriptional-activation experiment using *Arabidopsis* protoplasts, it was found that the transcriptional activation of AtDREB2A was significantly lower than that of AtDREB1A [10]. Furthermore, there was also no significant phenotypic difference between the transgenic *Arabidopsis* with constitutive expression of DREB2A and wild-type *Arabidopsis* [10].

DREB transcription factors bind with high specificity to the DRE/CRT cis-acting elements in promoters and thereby trans-activating the expression of these downstream target genes. Thus, DREB genes play an important role in regulating the signal pathways of plant-stress responses. Our experiments using a yeast one-hybrid system show that the HcDREB functioned as a transcriptional activator by specifically binding to a mutant DRE element. There are valine and glutamic acids at amino acid residues 14 and 19, respectively, of the AP2/EREBP domain of the HcDREB2. Both of these residues might play important roles in determining the specificity of the DNA-binding domain [10, 30]. However, as reported in previous studies in rice and barley, the valine at amino acid residue 14 of the AP2/EREBP region may have a decisive effect on the specificity of the DNA-binding domain [31, 32].

## Conclusions

We successfully cloned a putative DREB gene (HcDREB2) from *H. compressa* using the RACE-PCR method. The 1296-bp long HcDREB2 gene encoded a putative protein that consisted of 264 amino acid residues. HcDREB2 shared the highest sequence identity with the DREB2 gene in sorghum. The expression of HcDREB2 was independent of ABA, but was inducible by low temperature as well as drought and salinity (i.e., high NaCl concentration) treatments. The results of a yeast one-hybrid assay showed that HcDREB2 directly bound the DRE cis-acting element to transactivate the expression of the downstream reporter gene. Our study has established a foundation for future research in exploiting stress tolerance mechanisms and molecular breeding of *H. compressa* and other species.

## Methods

### Plant material

The *H. compressa* cultivar ’Guangyi’ was used in this study. The plants were maintained and propagated through vegetative cuttings: The mature stems (length >1 m) of *H. compressa* ‘Guangyi’ were selected and cut into two cuttings. The cuttings were inserted into 10-cm diameter plastic pots filled with soil, and one node of each cutting was buried in the soil. After shearing the leaves of nodes, one of every three cuttings was put into a conical flask (50 ml) with 25 ml of pure water. The conical flasks were put in a light growth chamber [28 °C/20 °C (day/night),12 h/12 h (day/night)] for 15 days before stress treatments.

### Stress treatments

After the cuttings generated new roots and leaves (about 15 days after transplanting), the conical flasks were transferred to another light growth chamber (4 °C). For drought, salinity, and ABA treatments, the culture water was supplemented with 20 % PEG6000, 250 mM NaCl, and 100 μM ABA, respectively. The tender leaves were sampled at 1, 2, 4, 6, 8, 12, and 24 h after treatments and were frozen in liquid nitrogen. Untreated plants were used as the controls.

### cDNA cloning of DREB

The total RNA was extracted from collected samples using the standard TRIzol protocol (Invitrogen, Carlsbad, CA, USA). First-strand cDNA was then synthesized using the PrimeScript™ RT reagent (Takara, Shiga, Japan) and a poly(T)-adaptor primer. An alignment of the DREB genes of model plants such as *Oryza sativa*, *Arabidopsis thaliana*, *Brachypodium distachyon*, revealed the AP2/ERF domain was relative conservative. Accordingly, based on the conserved AP2/ERF sequences, a cDNA fragment of DREB from the ’Guangyi’ cultivar was then PCR-amplified using the cDNA as template, and a degenerate primer pair (HcDREBF00 and HcDREBR00) was used to amplify the first fragment of the target gene from the cold-treated leaf samples (4 h after treatment; Additional file 2: Table S2). The PCR conditions were as follows: pre-heating at 94 °C for 3 min; 30 cycles at 94 °C for 30 s, 50 °C for 30 s and 72 °C for 1 min; and a final extension at 72 °C for 10 min. PCR products were separated by agarose gel electrophoresis, and the target DNA fragment was recovered using a gel extraction kit (Axygen, Corning, NY, USA). The recovered DNA fragments were then ligated into a pMD19-T vector (Takara). DNA fragments were sequenced at the Beijing Genomics Institute.

The full-length cDNA sequence was isolated using RACE-PCR. The 3’- and 5’- ends of the gene were amplified by using the 3’-Full RACE Core Set Ver.2.0 kit (Takara) and the 5’-Full RACE kit (Takara). For 3’-RACE PCR, the reaction conditions were as follows: pre-heating at 94 °C for 3 min; 30 cycles at 94 °C for 30 s, 50 °C for 30 s, and 72 °C for 1 min; and a final extension at 72 °C for 10 min. For 5’-RACE PCR, the reaction conditions were as follows: pre-heating at 94 °C for 3 min, 30 cycles at 94 °C for 30 s, 50 °C for 30 s, and 72 °C for 1 min; and a final extension at 72 °C for 10 min. DNA recovery and ligation were the same as described above.

### Gene transcription analysis

Total RNA extraction and first-strand cDNA synthesis were conducted as described above. The transcription level of the HcDREB2 gene relative to *β-actin* was determined by quantitative real-time reverse-transcriptase PCR (qRT-PCR). The qRT-PCR reactions were carried out in 96-well blocks in a BIO-RAD CFX96 Real-Time PCR system (Bio-Rad, Hercules, CA, USA). Each 20-μl reaction contained 2 μl of cDNA reaction mixture, 0.4 μl of ROX Reference Dye II, 10 μl of 2× SYBR® Premix Ex TaqTM (Takara), 0.4 μl of each primer (10 μM), and 6.8 μl of ddH2O. The reaction conditions were 30 s at 95 °C followed by 40 cycles of 95 °C for 5 s and 60 °C for 34 s. The dissociation curve was acquired by heating the amplicon from 60 °C to 95 °C. All qRT-PCR reactions were implemented in both technical and biological triplicates. In order to adjust results for background levels of gene expression, an internal control gene (*β-actin*) was used (Additional file 3). There are a number of common internal control genes used for qRT-PCR in plants, and these include 25 s ribosomal RNA (25S rRNA), glyceraldehyde 3-phosphate dehydrogenase (GAPDH), *β-actin*, and *β-tubulin* [33]. We used the 2^-△△Ct^ method [34] to calculate relative expression. And then SPSS (version 16.0; SPSS Inc., Chicago, IL USA) was used to conduct a one-way ANOVA, and a Duncan’s new multiple range test was used to test for differences in means of the expression data while controlling for multiple comparisons.

### Yeast one-hybrid system

We examined the transcriptional activation of HcDREB2 using the yeast one-hybrid system. The putative coding sequence of HcDREB2 was PCR-amplified (see above) and then inserted into a pGADT7-Rec AD cloning vector (Clontech, Mountain View, CA, USA) in order to construct the fusion-expression vector pGADT7-Rec-HcDREB. The wild-type and mutant-type of triple DRE cis-acting elements DREy and DREt, respectively, were separately inserted into the pAbAi vector to construct the fusion expression vectors DREy-pAbAi and DREt-pAbAi. The vectors pGADT7-Rec–HcDREB, DREy-pAbAi, and DREt-pAbAi were then together transformed into Y1H Gold yeast cells. The transformation pAbAi-p53 + pGADT7-Rec-p53 was used as a positive control, pAbAi + pGADT7-Rec was used as a negative control, and pAbAi + pGADT7-DREB was used as a blank control. Yeast cells with these vector combinations were inoculated onto SD plates under appropriate selection regimes for at least 48 h at 30 °C.

## Additional files

Additional file 1: Table S1. Primer used in isolating *HcDREB2* gene. (DOCX 16 kb)
Additional file 2: Table S2. Primer used in Real-Time PCR analysis. (DOCX 15 kb)
Additional file 3: Nucleotide sequence and deduced amino-acid sequence of HcDREB cDNA. (DOC 31 kb)

## Abbreviations

25S rRNA: 25 s ribosomal RNA
qRT-PCR: quantitative real-time reverse-transcriptase PCR
RACE-PCR: rapid amplification of cDNA end-PCR

## References

[1] Chen WJ, Zhu T (2004). Networks of transcription factors with roles in environmental stress response. Trends Plant Sci.

[2] Yamaguchi-Shinozaki K, Shinozaki K (2006). Transcriptional regulatory networks in cellular responses and tolerance to dehydration and cold stresses. Annu Rev Plant Biol.

[3] Elliott RC, Betzner AS, Huttner E, Oakes MP, Tucker WQ, Gerentes D (1996). AINTEGUMENTA, an APETALA2-like gene of Arabidopsis with pleiotropic roles in ovule development and floral organ growth. Plant Cell Online.

[4] Klucher KM, Chow H, Reiser L, Fischer RL (1996). The AINTEGUMENTA gene of Arabidopsis required for ovule and female gametophyte development is related to the floral homeotic gene APETALA2. Plant Cell Online.

[5] Zhuang J, Sun CC, Zhou XR, Xiong AS, Zhang J (2011). Isolation and characterization of an AP2/ERF-RAV transcription factor BnaRAV-1-HY15 in Brassica napus L. HuYou15. Mol Biol Rep.

[6] Zhuang J, Zhu B (2014). Analysis of Brassica napus ESTs: gene discovery and expression patterns of AP2/ERF-family transcription factors. Mol Biol Rep.

[7] Shinozaki K, Yamaguchi-Shinozaki K (2000). Molecular responses to dehydration and low temperature: differences and cross-talk between two stress signaling pathways. Curr Opin Plant Biol.

[8] Ohme-Takagi M, Suzuki K, Shinshi H (2000). Regulation of ethylene-induced transcription of defense genes. Plant Cell Physiol.

[9] Sakuma Y, Liu Q, Dubouzet JG, Abe H, Shinozaki K, Yamaguchi-Shinozaki K (2002). DNA-binding specificity of the ERF/AP2 domain of Arabidopsis DREBs, transcription factors involved in dehydration-and cold-inducible gene expression. Biochem Biophys Res Commun.

[10] Liu Q, Kasuga M, Sakuma Y, Abe H, Miura S, Yamaguchi-Shinozaki K (1998). Two transcription factors, DREB1 and DREB2, with an EREBP/AP2 DNA binding domain separate two cellular signal transduction pathways in drought-and low-temperature-responsive gene expression, respectively, in Arabidopsis. Plant Cell Online.

[11] Thomashow MF (1999). Plant cold acclimation: freezing tolerance genes and regulatory mechanisms. Annu Rev Plant Biol.

[12] Yamaguchi-Shinozaki K, Shinozaki K (1994). A novel cis-acting element in an Arabidopsis gene is involved in responsiveness to drought, low-temperature, or high-salt stress. Plant Cell Online.

[13] Haake V, Cook D, Riechmann JL, Pineda O, Thomashow MF, Zhang JZ (2002). Transcription factor CBF4 is a regulator of drought adaptation in Arabidopsis. Plant Physiol.

[14] Liu WQ, Shi YC, Hu YJ, Liu QZ (2007). The tolerance to abiotic stresses mediated by DREB-like transcription factors in Nicotiana tabacum. Journal of Wuhan Botanical Research.

[15] Chen PH, Chen RX, Xu LP, Wang HB, Chen LP, Lin B (2011). Whole genome amplification of single pollen grains from a sugarcane cutivar and anaylysis of the genetic relatedness based on SCoT Markers. Chin J Trop Crop.

[16] Tang MJ, Lü SY, Jing YX, Zhou XJ, Sun JW, Shen SH (2005). Isolation and identification of a cold-inducible gene encoding a putative DRE-binding transcription factor from Festuca arundinacea. Plant Physiol Biochem.

[17] Xiong YW, Fei SZ (2006). Functional and phylogenetic analysis of a DREB/CBF-like gene in perennial ryegrass (Lolium perenne L.). Planta.

[18] Niu YD, Hu TM, Zhou YG, Hasi A (2010). Isolation and characterization of two Medicago falcate AP2/EREBP family transcription factor cDNA, MfDREB1 and MfDREB1s. Plant Physiol Biochem.

[19] Dubouzet JG, Sakuma Y, Ito Y, Kasuga M, Dubouzet EG, Miura S (2003). OsDREB genes in rice, Oryza sativa L., encode transcription activators that function in drought‐, high‐salt and cold‐responsive gene expression. Plant J.

[20] Liu LQ, Zhu K, Yang YF, Wu J, Chen FD, Yu DY (2008). Molecular cloning, expression profiling and trans-activation property studies of a DREB2-like gene from chrysanthemum (Dendranthema vestitum). J Plant Res.

[21] Qin F, Kakimoto M, Sakuma Y, Maruyama K, Osakabe Y, Tran LSP (2007). Regulation and functional analysis of ZmDREB2A in response to drought and heat stresses in Zea mays L. Plant J.

[22] Medina JN, Bargues M, Terol J, Pérez-Alonso M, Salinas J (1999). The Arabidopsis CBF gene family is composed of three genes encoding AP2 domain-containing proteins whose expression is regulated by low temperature but not by abscisic acid or dehydration. Plant Physiol.

[23] Nakashima K, Shinwari ZK, Sakuma Y, Seki M, Miura S, Shinozaki K (2000). Organization and expression of two Arabidopsis DREB2 genes encoding DRE-binding proteins involved in dehydration and high-salinity-responsive gene expression. Plant Mol Biol.

[24] Shen YG, Zhang WK, He SJ, Zhang JS, Liu Q, Chen SY (2003). An EREBP/AP2-type protein in Triticum aestivum was a DRE-binding transcription factor induced by cold, dehydration and ABA stress. Theor Appl Genet.

[25] Xue GP, Loveridge CW (2004). HvDRF1 is involved in abscisic acid‐mediated gene regulation in barley and produces two forms of AP2 transcriptional activators, interacting preferably with a CT‐rich element. Plant J.

[26] Clifton-Brown JC, Lewandowsk I, Andersson B, Basch G, Christian DG (2007). Stress-inducible DREB2A transcription factor from Pennisetum glaucum is a phosphoprotein and its phosphorylation negatively regulates its DNA-binding activity. Mol Genet Genomic.

[27] Xie YL, Wang ZZ, Liu Q, Zhang SP (2006). Cloning and functional identification of stress-resistant BeDREB genes from Bermuda grass. Front Biol Chin.

[28] Fu X, Peng RH, Zhang Z, Qiao YS, Zhou J, Zhu B (2009). Cloning and expression of transcription factor gene MrDREBA6 from Malus micromalus. J Fruit Sci.

[29] Fowler SG, Cook D, Thomashow MF (2005). Low temperature induction of Arabidopsis CBF1, 2, and 3 is gated by the circadian clock. Plant Physiol.

[30] Sakuma Y, Maruyama K, Osakabe Y, Qin F, Seki M, Shinozaki K (2006). Functional analysis of an Arabidopsis transcription factor, DREB2A, involved in drought-responsive gene expression. Plant Cell Online.

[31] Sharoni AM, Nuruzzaman M, Satoh K, Shimizu T, Kondoh H, Sasaya T (2011). Gene structures, classification and expression models of the AP2/EREBP transcription factor family in rice. Plant Cell Physiol.

[32] Skinner JS, von Zitzewitz J, Szűcs P, Marquez-Cedillo L, Filichkin T, Amundsen K (2005). Structural, functional, and phylogenetic characterization of a large CBF gene family in barley. Plant Mol Biol.

[33] Iskandar HM, Simpson RS, Casu RE, Bonnett GD, Maclean DJ, Manners JM (2004). Comparison of reference genes for quantitative real-time polymerase chain reaction analysis of gene expression in sugarcane. Plant Mol Biol Rep.

[34] Zhu H-T, Dong Q-Z, Wang G, Zhou H-J, Ren N, Jia H-L (2012). Identification of suitable reference genes for qRT-PCR analysis of circulating microRNAs in hepatitis B virus-infected patients. Mol Biotechnol.

