# Supplementary material for: Cloning and characterization of an ABA-independent DREB transcription factor gene, HcDREB2, in Hemarthria compressa
Source: Hereditas. 2016 Apr 8;153:3. doi: 10.1186/s41065-016-0008-y (PMC5224587; doi:10.1186/s41065-016-0008-y)
Supplement: Additional file 1: Table S1. — Primer used in isolating HcDREB2 gene. (DOCX 16 kb) [file 41065_2016_8_MOESM1_ESM.docx]

**Additional file 1: Table S1.** Primer used in isolating *HcDREB2* gene.

| **Objective** | **Forward primers** | **Primer sequence** | **Reverse primers** | **Primer sequence** |
| --- | --- | --- | --- | --- |
| Amplifiy conservative domain | HcDREBF00 | GGKTCCAAGAAAGGKTGC | HcDREBR00 | BGAGAARAGRCTAAAMCCRTC |
| Amplify 3` terminal of cDNA | HcDREBF00 | GGKTCCAAGAAAGGKTGC | 3’RACE Outer Primer |  |
|  | HcDREBF01 | CTTTCGTTGCTGGCATCTAGTGT | 3’RACE Inner Primer |  |
| amplify 5` terminal of cDNA | HcDREBR03 | TAGATGCCAGCAACGAAAGCG | 5’RACE Outer Primer |  |
|  | HcDREBR02 | CAGCCATAGCCTGCGACCAC | 5’RACE Inner Primer |  |
| Amplifiy gene full length | HcDREBF04 | AACGAGGATACCACCGCCACCG | HcDREBR04 | GTCTGAGGCACAAAAAGTATG |

Note: K = (G,T); M = (A,C); B = (C,G,T); R = (A,G).
