# Supplementary material for: Cloning and characterization of an ABA-independent DREB transcription factor gene, HcDREB2, in Hemarthria compressa
Source: Hereditas. 2016 Apr 8;153:3. doi: 10.1186/s41065-016-0008-y (PMC5224587; doi:10.1186/s41065-016-0008-y)
Supplement: Additional file 2: Table S2. — Primer used in Real-Time PCR analysis. (DOCX 15 kb) [file 41065_2016_8_MOESM2_ESM.docx]

**Additional file 2: Table S2.** Primer used in Real-Time PCR analysis.

| **Forward primers** | **Primer sequence** | **Reverse primers** | **Primer sequence** |
| --- | --- | --- | --- |
| β-actin L | CCCAATCTATGAAGGCTACGC | β-actin R | CGGCAGTGGTTGTGAAAGAGTA |
| HcDERBL05 | CTGACTCCATTGCTGAGACGAT | HcDERBR05 | GGTACACGCAGTTGACGTTCTC |
