# Supplementary material for: Cloning and characterization of an ABA-independent DREB transcription factor gene, HcDREB2, in Hemarthria compressa
Source: Hereditas. 2016 Apr 8;153:3. doi: 10.1186/s41065-016-0008-y (PMC5224587; doi:10.1186/s41065-016-0008-y)
Supplement: Additional file 3: — Nucleotide sequence and deduced amino-acid sequence of HcDREB cDNA. (DOC 31 kb) [file 41065_2016_8_MOESM3_ESM.doc]

1 AGAGGACGGAGGAGGAGCAGGGAGATCTCTCGCTCTCGCTCCCTTCTCGCTCTTCCGTCC

61 ATGGAGCTCGGAGACGCCGCCGCCGCCGCCGCCCAGGGAGGACCGCAAGGGGACGCCTCG

21 M E L G D A A A A A A Q G G P Q G D A S

121 GGGGCCCTTGTCAGGAAGAAGAGGATGAGGAGGAAGAGCTCTGGCCCTGACTCCATTGCC

41 G A L V R K K R M R R K S S G P D S I A

181 GAGACGATCAAGTGGTGGAAGGAGCAGAACCAGAAGCTGCAGGACGAGAGTGGCTCCAGG

61 E T I K W W K E Q N Q K L Q D E S G S R

241 AAGGCGCCAGCCAAGGGTTCCAAGAAAGGGTGCATGACGGGCAAAGGAGGGCCTGAGAAC

81 K A P A K G S K K G C M T G K G G P E N

301 GTCAACTGCGTGTACCGCGGCGTCAGGCAGCGGACGTGGGGCAAGTGGGTGGCGGAGATC

101 V N C V Y R G V R Q R T W G K W V A E I

361 CGCGAGCCCAACCGTGGTCGCAGGCTATGGCTGGGCTCCTTCCCTACTGCTGTGGAGGCT

121 R E P N R G R R L W L G S F P T A V E A

421 GCCCATGCATACGATGAGGCGGCAAAGGCGATGTATGGCCCCAGGGCACGTGTCAACTTC

141 A H A Y D E A A K A M Y G P R A R V N F

481 TCGGAGAACTCTGCTGACGCTAACTCTGGCTGCACGTCGGCGCTTTCGTTGCTGGCATCT

161 S E N S A D A N S G C T S A L S L L A S

541 AGTGTGCCGGCTGCCACGTTGCAGCGGTCTGATGAGAAAGTGGAGACTGAGGTGGAATCT

181 S V P A A T L Q R S D E K V E T E V E S

601 GTGGAGACTGGGGTCCATGAGGTGAAAACAGAAGCGAATGATGACTTGGTAAGTGTCCAC

201 V E T G V H E V K T E A N D D L V S V H

661 ATTGCCTGCAAGACTGTGGACGTCATTCAGCCCGAGAAGAGTGTCTTACACAAGGAAGTG

221 I A C K T V D V I Q P E K S V L H K E V

721 GACATAAGTTATGATTACTTCAACGTCGAAGAGGTGGTTGAGATGATAATTATAGAATTG

241 D I S Y D Y F N V E E V V E M I I I E L

781 AATGCTGATAAAAAAATCGAAGCACATGAAGAATACCATGATGGAGATGATGGGTTTAGC

261 N A D K K I E A H E E Y H D G D D G F S

841 CTTTTTGCATATTAGAAGCATGGTAATGGGGAACAGTAGGAATAACTTCATTCTAGATGT

281 L F A Y *

901 TAGGAAAACACTTCAACCTGAAGCGTTGTAGTCATTCGTGGTTTTCATCTTACTGAGACA

961 TAGCTTTATACTATGAGCCAACCGGTACAAGAAGTTGTCCTGTGTGTTGAGTTCCTGTAC

1021 TATAGTAGGAAATGAGTCCGTGTTTAATGAGCTCTCTTGGTTGTTAATATTGCACATTTG

1081 CTCGGGGGTGAACTCAAGTTAGCTCAAGAAAAGGAATGGTAAGTACACCGAATAAATTTT

1141 TAGGATTGCATTTAATTGCAAAGCAAGCATATGTTATTTCGATGTAACAGTACATTCAGT

1201 TACAATAGAGTATCATACTTTTTGTGCCTCAGACAATCGTCGACCTGCAGGCAT

**Additional file 3 Nucleotide sequence and deduced amino-acid sequence of HcDREB cDNA.**

The letters with thick underline represent AP2 domain; The letters with double underline represent the core amino acid of nuclear localization signals(NLS); The letters with dotted underline represent transcriptional activation domain; * - Stop-codon.
